# RESILIENCY AMONG WHI WOMEN IN THE 80+ COHORT BY RACE AND NEIGHBORHOOD SOCIOECONOMIC STATUS

**DOI:** 10.1093/geroni/igac059.367

**Published:** 2022-12-20

**Authors:** Jessica Krok-Schoen, Michelle Naughton, Crystal Cene, Aladdin Shadyab, Sparkle Springfield, Timiya Nolan, Ashley Felix, Rebecca Jackson

**Affiliations:** The Ohio State University, Columbus, Ohio, United States; The Ohio State University Comprehensive Cancer Center, Columbus, Ohio, United States; University of North Carolina, Chapel Hill, North Carolina, United States; University of California at San Diego, La Jolla, California, United States; Loyola University, Maywood, Illinois, United States; The Ohio State University, Columbus, Ohio, United States; The Ohio State University, Columbus, Ohio, United States; The Ohio State University, Columbus, Ohio, United States

## Abstract

Resilience, an individual’s ability to successfully adapt to adversity, is a multifaceted outcome that may be affected by individual and community factors. A comprehensive examination of resilience by race and neighborhood socioeconomic status (NSES) among women aged 80+ is needed to better understand longevity in diverse populations. Women aged 80+ in 2011, in the Women’s Health Initiative (WHI) study were included. Resilience was measured using the 3-item Brief Resilience Scale, with higher scores indicating better resiliency. Descriptive statistics and multivariable linear regression examined the association of demographic, psychosocial, and health variables with resilience by race (White, Black, Asian) and NSES. The majority of participants (n=29,367, median age=84.0) were non-Hispanic White (91.4%), and had multimorbidities (66%). There were no significant differences by race on mean resiliency scores (p=0.06). Mean resilience was higher among women with higher NSES (low NSES=3.94±0.83, moderate NSES=3.95±0.82, high NSES=4.00±0.81; p< 0.001). Optimism (p< 0.001), social support (p< 0.01), and physical/mental symptom burden (p< 0.05) were significant correlates of resilience among Asian, Black, and White women. Self-rated health (p< 0.001), depressive symptoms (p< 0.001), optimism (p< 0.001), social support (p< 0.001), physical/mental symptom burden (p< 0.001), and body mass index (p< 0.001) were significant correlates of resilience across women with low, moderate, and high NSES. Age was significantly associated with resilience among women with moderate (β=-0.004, p=0.019) and high NSES (β=-0.005, p=0.045). This study found several common correlates of resilience across race and NSES among women aged 80+ in the WHI. Future research to enhance resilience, such as through psychosocial and behavioral interventions, is warranted.

